# PEGylation of liposome-encapsulated midazolam does not improve the bioavailability of midazolam when administered orally

**DOI:** 10.1186/s40360-025-00993-1

**Published:** 2025-10-15

**Authors:** Yukiko Nishioka, Yanyin Lu, Hitoshi Higuchi, Saki Miyake, Maki Fujimoto, Midori Hamaoka-Inoue, Hiroshi Tanimura, Hitomi Ujita, Shigeru Maeda, Takuya Miyawaki

**Affiliations:** 1 Department of Dental Anesthesiology, Okayama University Hospital, 2-5-1 Shikata-cho, Kita-ku, Okayama, 700-8525 Japan; 2 Department of Dental Anesthesiology and Special Care Dentistry, Okayama University Graduate School of Medicine, Dentistry and Pharmaceutical Sciences, 2-5-1 Shikata-cho, Kita-ku, Okayama, 700-8525 Japan; 3 Present Address: Department of Dental Anesthesiology, Graduate School of Medical and Dental Sciences, Institute of Science Tokyo, 1-5-45 Yushima, Bunkyo-ku, Tokyo, 113-8549 Japan

**Keywords:** PEGylation, Liposome, Midazolam, Oral administration, Bioavailability

## Abstract

**Background:**

Liposomes are closed vesicles made of the same phospholipid bilayer as biological membranes and are capable of containing drugs, and so they have been investigated as useful drug carriers for drug delivery. We previously developed liposome-encapsulated midazolam (LE-midazolam) for oral administration, but midazolam is metabolized in the liver, and for clinical use the encapsulation of the liposomes needed to be improved to increase the bioavailability of midazolam. The surfaces of pharmaceutical liposomes are generally coated with polyethylene glycol (PEGylation) because it prevents their capture by phagocytes and helps them to avoid the reticuloendothelial system. Therefore, we considered that PEGylation could reduce the metabolism of orally administered encapsulated midazolam in the liver.

**Methods:**

Midazolam solution, LE-midazolam solution, and PEGylated liposome-encapsulated midazolam (PEG-LE-midazolam) solution were prepared, and the characteristics of the liposomes in these solutions were evaluated. Furthermore, these solutions were orally administered to rabbits, and the resultant plasma midazolam concentrations were measured. The effects of the PEGylation of LE-midazolam on the plasma concentration and bioavailability of orally administered midazolam were also evaluated.

**Results:**

The PEG-LE-midazolam solution contained a higher percentage of larger liposomes than the LE-midazolam solution. The area under the concentration-time curve (AUC) of the LE-midazolam solution was significantly higher than that of the midazolam solution, but there was no difference between the AUC values of the PEG-LE-midazolam and midazolam solutions.

**Conclusions:**

These findings suggest that liposome encapsulation may reduce the first-pass effect following oral administration, but PEGylation is not expected to improve the bioavailability of orally administered midazolam.

**Supplementary Information:**

The online version contains supplementary material available at 10.1186/s40360-025-00993-1.

## Introduction

Liposomes are closed vesicles made of the same phospholipid bilayer as biological membranes and are capable of containing drugs, including both water- and lipid-soluble compounds [1]. Since liposomes are comprised of biological components and exhibit superior biocompatibility and in vivo degradation abilities, they have been investigated as useful drug carriers in the drug delivery system field [1–3].

Midazolam, an intravenous anesthetic, is frequently used for intravenous anesthesia and sedation. It is also used for premedication because it has anxiolytic effects, e.g., it is used to reduce anxiety and stress in pediatric and intellectually disabled patients [4]. There are various administration routes for midazolam, but intravenous and intramuscular administration are painful. Nasal administration results in better absorption and is not subject to first-pass effects, but the low pH and high concentration of such preparations can have adverse effects on the nasal mucosa. Rectal administration is affected by the degree of drug absorption and defecation, and is also more stressful. Oral administration is more useful than other administration routes because it is less painful, has fewer adverse effects, and is less stressful for patients with anxiety. However, due to the strong bitter taste of midazolam, oral administration is difficult in pediatric and intellectually disabled patients. There is no clinically indicated midazolam syrup available in Japan. Therefore, midazolam solution is administered by adding syrup or water to reduce its bitterness. Thus, we previously developed liposome-encapsulated midazolam (LE-midazolam) for oral administration to reduce its bitter taste [5].

There has been much research into the development of liposome-encapsulated drugs, such as insulin [6] and griseofulvin [7], for oral administration, especially liposome-encapsulated insulin for oral administration. Over the past decade, various materials have been used to improve encapsulation efficiency, drug absorption in the intestinal tract, and bioavailability [8]. However, the optimal components for liposomes designed to contain drugs other than insulin for oral administration have not been fully elucidated. It is considered that this depends on the properties of each drug; therefore, we need to resolve basic issues, including the optimal materials and particle size for liposomes designed for oral drug administration. Our previous study showed that the plasma concentration of midazolam was significantly higher in rabbits when liposome-encapsulated solution was administered orally than when midazolam solution was administered orally. Midazolam is a typical benzodiazepine and is metabolized by cytochrome P450 (CYP). CYPs are distributed in the intestinal tract and play a major role in the metabolism of orally administered drugs. Therefore, liposomes may affect not only the absorption of midazolam, but also its metabolism by CYPs in the intestinal tract, resulting in higher bioavailability of LE-midazolam. Basic research into liposomes for oral drug administration is required to increase the bioavailability of drugs during clinical use.

Polyethylene glycol (PEG) is a polymer composed of ethylene glycol. Liposomes are captured by phagocytes, which causes problems with their stability and retention in the circulation [9], but coating the surfaces of liposomes with PEG (PEGylation) prevents phagocytes and opsonin from interacting with them, resulting in them avoiding the reticuloendothelial system (RES) [10, 11]. Therefore, pharmaceutical liposomes are generally subjected to PEGylation. We considered that PEGylation may reduce the metabolism of encapsulated midazolam in the liver, even when it is administered orally. Furthermore, PEGylation may promote escape from the first-pass effect in the intestinal tract and reduce capture by the RES to some extent [12, 13]. Therefore, in the present study we evaluated the effects of the PEGylation of LE-midazolam on the plasma concentration and bioavailability of orally administered midazolam. Furthermore, the plasma concentration of phosphatidylcholine, which is used to make liposomes, was measured to evaluate the release of liposomes from the intestinal tract into the bloodstream, and the difference in the plasma concentration of midazolam between intravenously administered midazolam and intravenously administered LE-midazolam was also assessed.

## Methods

### Reagents

L-α-phosphatidylcholine (PCHL); cholesterol; dimyristoylphosphatidylcholine (DMPC); L-α-phosphatidylethanolamine, distearoyl methoxypolyethylene glycol conjugate; and potassium phosphate were purchased from Sigma (St. Louis, USA). Midazolam; diazepam, which was used as an internal standard (IS) for measuring the midazolam concentration using high-performance liquid chromatography (HPLC); and potassium dihydrogen phosphate solution were obtained from Wako Pure Chemical Industries (Osaka, Japan). HPLC-grade distilled water, methanol, chloroform, hydrochloric acid solution, and Tris-hydrochloride buffer were purchased from Nacalai Tesque (Tokyo, Japan). Acetonitrile and diethyl ether were purchased from Kanto Chemical Co., Inc. (Tokyo, Japan). Isoflurane was purchased from Abbvie GK (Tokyo, Japan).

### Preparation of midazolam-encapsulating liposomes

According to the method of Tomoyasu et al., PCHL, cholesterol, DMPC, and midazolam were diluted with a chloroform/methanol solvent mixture (chloroform : methanol = 2 : 1) and mixed at a molar ratio of PCHL : cholesterol : DMPC : midazolam = 1 : 1 : 0.1 : 0.5. For the PEGylated liposomes, PEG was added to the mixture to give a concentration of 9 mol% of the total lipid molar mass. Each mixture with or without PEG was evaporated using an evaporator at a bath temperature of 45℃ to prepare a lipid film. After being dried using a vacuum pump for one hour, the lipid film was combined with 2 mL of 0.1 N hydrochloric acid solution and shaken in a water bath at 50℃ to suspend liposome particles from the lipid film. Then, 7.5 mL of 0.2 M Tris-hydrochloride buffer (pH 7.6) was added to 0.5 mL of the liposome suspension and separated by ultracentrifugation (TX-160 TOMY) at 15,000 g for 20 min at 4℃. The supernatant containing extra buffer and the unencapsulated drug was removed, and 0.5 mL of 0.2 M Tris-hydrochloride buffer (pH 7.6) was added to the precipitate to create the pre-homogenized liposome solution. This liposome solution was placed in an ultrasonic homogenizer (Bioruptor, UCD-200TM, Cosmo Bio, Tokyo, Japan) (200 W, 20 kHz, and 10℃), and the liposome particles were homogenized for 20 min to form LE-midazolam solution and PEGylated LE-midazolam (PEG-LE-midazolam) solution. The particle size (liposome diameter) distribution, volume share rate of each peak size distribution, and zeta potential of the liposomes in these solutions were measured. The mean diameter of the vesicles and the polydispersity index were determined by dynamic light scattering (DLS) at 25 °C and a scattering angle of 173°. The zeta potential was determined based on the electrophoretic mobility according to DLS. For the particle size measurements, 60 µL of liposome solution was diluted with 60 µL of 0.2 M Tris-hydrochloride buffer, and for the zeta potential measurements 150 µL of liposome solution was diluted with 700 µL of distilled water. All samples were measured three times using a Zetasizer Nano ZSP instrument (Malvern Instruments, Worcestershire, UK).

### Determination of the Midazolam concentration and liposome encapsulation efficiency

According to the method of Tomoyasu et al., the midazolam concentration was determined by HPLC using the TSK gel ODS-80Ts column (Tosoh, Tokyo, Japan) and a mixture of acetonitrile, distilled water, and 0.25 M potassium dihydrogen phosphate in a ratio of 25:48:2 as the HPLC mobile phase, which was maintained at a flow rate of 1.0 mL/min. An ultraviolet absorption spectrophotometer with a wavelength of 214 nm was used to obtain the measurements. The IS method was used to determine the midazolam concentration, and the ratio of the peak area of midazolam to that of the IS (diazepam) was calculated. A 100 µL sample was diluted to 1 mL by adding 50% methanol, and then 50 µL of diazepam 100 µg/mL was added as an IS, from which 50 µL was withdrawn and injected into the HPLC. To determine the concentration of midazolam in a sample, a standard curve was prepared as follows. To 100 µL of midazolam reagent at each concentration (12.725–50.9 µg/mL) diluted in distilled water, 500 µL of 100% methanol and 400 µL of distilled water were added, and 50 µL of 100 µg/mL diazepam as IS was added thereto before analysis by HPLC. An equation calculated by linear regression analysis using the least-squares method was obtained from the ratio of the peak area of midazolam to the peak area of diazepam, and the concentration of midazolam in the sample was calculated from this equation for quantitative analysis. The liposome encapsulation efficiency was calculated with the formula: EE%=Ce/C0 × 100 (C0: the concentration of the liposome solution before centrifugation; Ce: the concentration of precipitated liposomes after centrifugation).

### Animals for the in vivo experiments

The in vivo experiments were conducted according to the method of Tomoyasu et al. Thirteen clean male New Zealand White rabbits, aged 10–11 weeks and weighing 2.19 ± 0.10 kg (1.96–2.34 kg) (SLC, Hamamatsu, Japan), were used for the oral administration experiment. Furthermore, 12 clean male New Zealand White rabbits, aged 10–11 weeks and weighing 2.11 ± 0.15 kg (1.95–2.42 kg) (SLC, Hamamatsu, Japan), were used for the intravenous administration experiment. The experiments were performed after approval was obtained from the Animal Experiment Management Committee of Okayama University (approval number: OKU-2018069) and were carried out in strict accordance with the recommendations outlined in the Guide for the Care and Use of Laboratory Animals at Okayama University and the International Guiding Principles for Biomedical Research Involving Animals by the International Council for Laboratory Animal Science. They were performed after a one-week acclimation period, in which the rabbits were fed pellets and water in an animal room controlled at a room temperature of 25℃. The pellets were changed to AIN93M (Oriental Yeast, Tokyo, Japan), in which cellulose was replaced by alfalfa (dietary fiber: 30.0% or less), from 3 days before the experiments, and the animals were fed only water and fasted for 18 h prior to use.

### Oral administration of the test solutions to rabbits and collection of plasma samples

Rabbits were anesthetized via the inhalation of 2.5 to 3.5% isoflurane, and a catheter was inserted into the femoral artery to continuously collect peripheral arterial blood samples. A 6.5-Fr stomach tube with a target length of 14 cm was inserted through the nose in order to administer the test solutions. The final stomach tube length was determined by listening for the sound of air being sent into the stomach using a stethoscope. Arousal of the rabbits was confirmed more than 60 min after the completion of the isoflurane-induced inhalation anesthesia based on leg movement and eye opening, and then 10 mL of the midazolam solution, LE-midazolam solution, or PEG-LE-midazolam solution was administered through the stomach tube. The midazolam solution was prepared by dissolving midazolam powder in 0.1 N hydrochloric acid solution, then in saline, and finally in Tris-hydrochloride buffer. The dose of midazolam in the test solutions was set at 2 mg/kg, and the pH of the solutions was set at around 7.0 for all preparations. The test solutions were administered through the stomach tube for 60 s, before 2 mL of saline was administered for 10 s to push out any test solution remaining in the tube.

Peripheral arterial blood (1.0 mL each) was collected from the femoral artery at the baseline (before the administration of the test solution) and at 5, 10, 20, 30, 60, 90, 120, 180, and 240 min after the administration of the test solution, centrifuged at 1,500 g for 10 min at room temperature, and then the separated plasma samples were stored at -30℃.

After the experiment, the rabbits were euthanized via the intravenous administration of 45 mg/kg propofol, which was approved by the Okayama University Animal Experiment Control Committee.

### Intravenous administration of the test solutions to rabbits and collection of plasma samples

Rabbits were prepared in the same manner as in the oral administration study described above. The midazolam solution was prepared by dissolving midazolam powder in 0.1 N hydrochloric acid solution, then in saline, and finally in Tris-hydrochloride buffer. The dose of midazolam in the test solutions was set at 0.2 mg/kg, and the pH of the solutions was set around 7.0 for all preparations. The initial non-homogenized liposome solution was used as the LE-midazolam solution. A bolus dose of each solution was administered to the rabbits through the auricular vein. Peripheral arterial blood (1.0 mL each) was collected from the femoral artery at the baseline (before the administration of the test solution) and at 5, 10, 15, 20, 25, 30, 45, 60, 90, and 180 min after the administration of the test solution. The midazolam concentration of each plasma sample was determined using HPLC in the same manner as in the oral administration study described above.

### Measurement of the plasma concentrations of Midazolam and phosphatidylcholine of the samples

The midazolam concentrations of the plasma samples collected before at the baseline and at 5, 10, 20, 30, 60, 90, 120, 180, and 240 min after the administration of the test solution were determined using HPLC. In the pretreatment stage, 0.5 mL of the plasma sample was combined with 0.5 mL of saline, 1 mL of 0.1 M dipotassium phosphate solution, 0.1 mL of the IS (2 µg/mL diazepam), and 5 mL of diethyl ether; shaken at 120/min for 10 min; and centrifuged at 1,400 g for 10 min, and then the ether layer was collected. The ether layer was combined with 4 mL of distilled water, shaken at 120/min for 10 min, re-centrifuged at 1,400 g for 10 min, and then the ether layer was collected again. The ether was evaporated to dryness, and the residue was resuspended in 200 µL of a mixture of methanol : acetonitrile : 0.01 M dipotassium phosphate = 2 : 9 : 9, and 50 µL of this solution was applied to HPLC.

The phosphatidylcholine concentrations of the plasma samples collected at the baseline and at 20 and 60 min after the administration of the test solution from the rabbits treated with LE-midazolam solution or PEG-LE-midazolam solution were determined using a phosphatidylcholine colorimetric assay kit (Cayman Chemical Company, Ann Arbor, USA).

### Bioavailability of orally administered test solutions

The bioavailability of the test solutions was calculated by dividing the area under the concentration-time curve (AUC) of plasma midazolam concentration after oral administration by the AUC of plasma midazolam concentration up to 180 min after intravenous administration of midazolam solution, and then multiplying the result by the ratio of the oral dose to the intravenous dose, expressed as a percentage.

### Statistical analysis

The significance of differences was analyzed in each experiment by employing the unpaired t test or one-way analysis of variance (ANOVA) followed by Tukey’s post-hoc multiple comparisons test using statistical analysis software (PRISM^®^ Version 4.0c, GraphPad Software, San Diego, USA), and p-values less than 5% (*P* < 0.05) were regarded as significant. The results are presented as the mean ± standard deviation (SD).

## Results

### Characteristics of liposomes

**Fig. 1:**
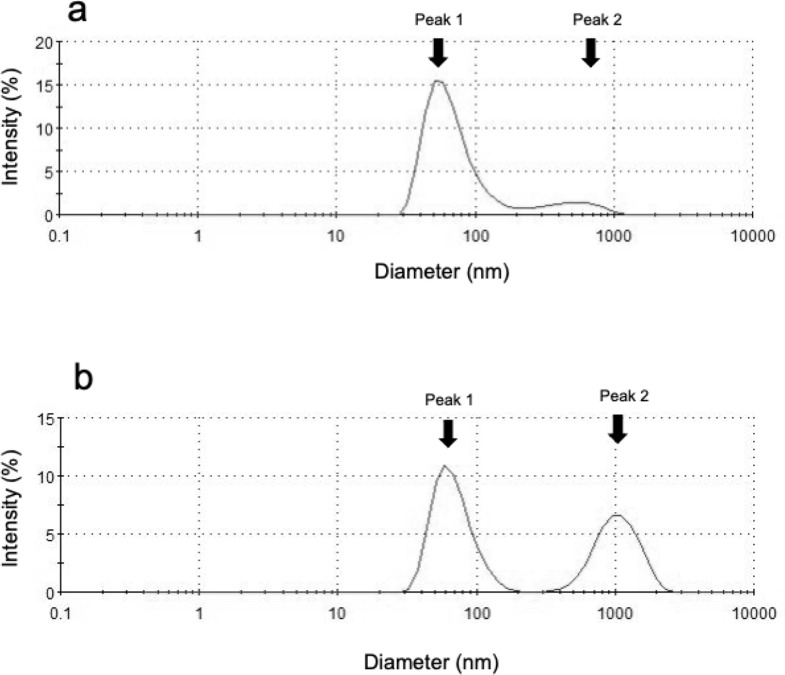
Typical size distribution of liposome particles in the test solutions. **a** Liposome-encapsulated midazolam solution (LE-midazolam). **b** PEGylated liposome-encapsulated midazolam solution (PEG-LE-midazolam)

Table 1 shows the characteristics of the liposomes in each solution. There was no significant difference in the midazolam-encapsulating efficiency of the liposomes between the LE-midazolam and PEG-LE-midazolam solutions. Figure 1 shows the typical size distribution of the liposome particles in the test solutions. The size distribution of the liposome particles in the test solutions showed two peaks. The first peak was around 70 nm in diameter for both solutions, but the percentage volume of the first peak in the size distribution of the PEG-LE-midazolam solution was lower than that of the LE-midazolam solution (*P* < 0.0001). The second peak was located at > 1,000 nm for the PEG-LE-midazolam solution whereas it was located around 400 nm for the LE-midazolam solution (*P* < 0.0001), and the percentage volume of the second peak in the size distribution was around 50% for the PEG-LE-midazolam solution, whereas it was less than 10% for the LE-midazolam solution (*P* < 0.0001). The zeta potential of the liposomes in the PEG-LE-midazolam solution was closer to zero than that of the liposomes in the LE-midazolam solution (*P* < 0.0001).

**Table 1:** Characteristics of liposomes in the test solutions

		LE-midazolam	PEG-LE-midazolam	*P*-values
Midazolam-encapsulating efficiency (%)		85.3 ± 4.6	83.4 ± 1.2	NS
First peak in size distribution of liposome particles	Particle size (nm)	68.1 ± 2.0 (65.6 to 70.5)	69. 8 ± 0.8 (68.7 to 70.6)	NS
	Volume (%)	91.0 ± 1.7 (88.8 to 92.8)	51.3 ± 5.7 (43.3 to 58.1)	< 0.0001
Second peak in size distribution of liposome particles	Particle size (nm)	401.1 ± 110.7 (272.0 to 514.0)	1,367.0 ± 197.0 (1075.0 to 1,553.0)	< 0.0001
	Volume (%)	9.0 ± 1.7 (7.2 to 11.2)	48.7 ± 5.7 (41.9 to 55.7)	0.003
Zeta potential of liposomes (mV)		-13.78 ± 0.56 (-14.3 to -12.7)	-4.58 ± 0.06 (-4.69 to -4.49)	< 0.0001

LE-midazolam: liposome-encapsulated midazolam solution

PEG-LE-midazolam: PEG-modified liposome-encapsulated midazolam solution

Analyzed using the unpaired t test

NS: Not significant

### Plasma Midazolam concentration after the oral administration of the test solutions

**Fig. 2:**
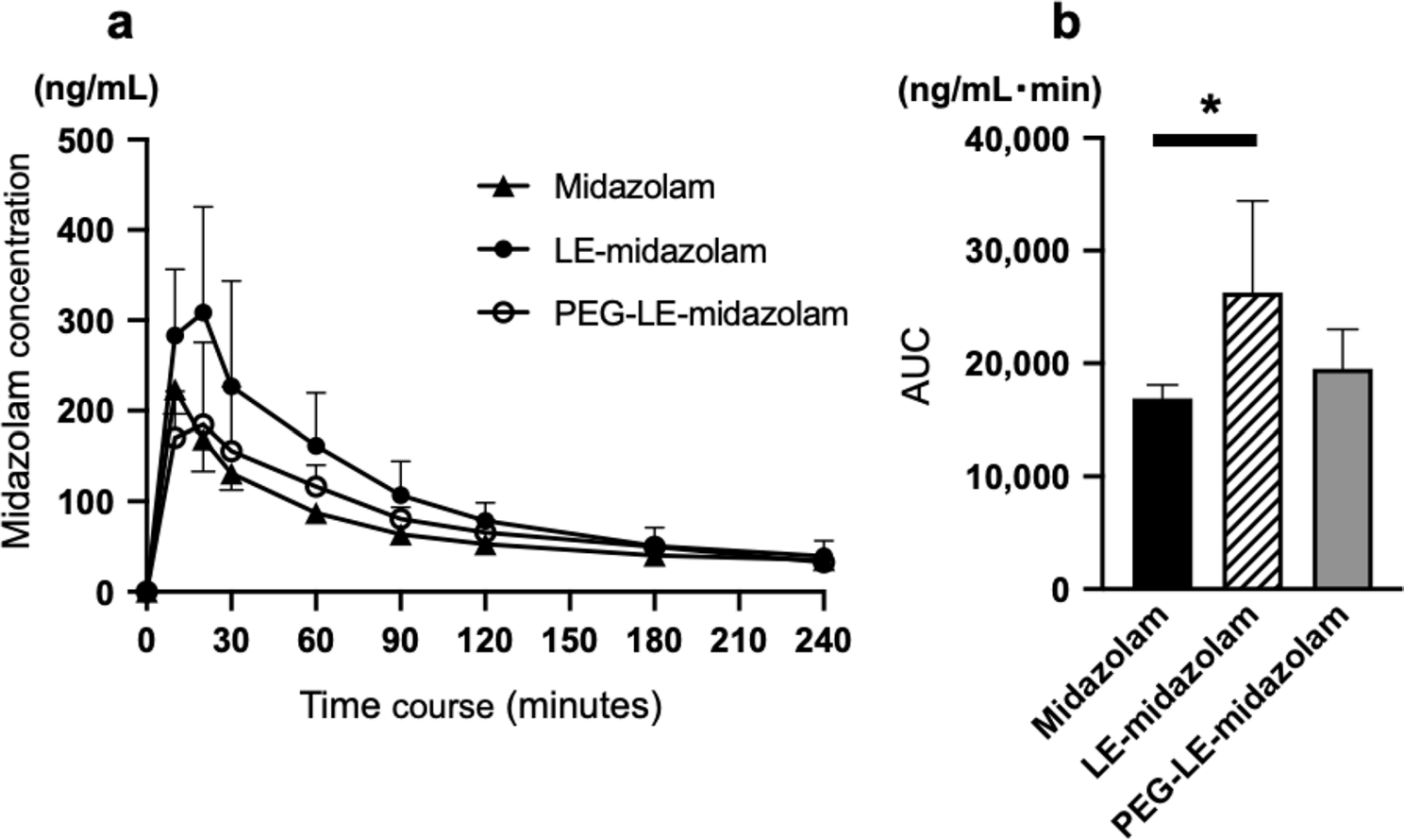
Plasma midazolam concentrations after the oral administration of the test solutions. **a** Time courses of the changes in the plasma midazolam concentration seen after the oral administration of midazolam (*n* = 4), liposome-encapsulated midazolam solution (LE-midazolam) (*n* = 5), or PEGylated liposome-encapsulated midazolam solution (PEG-LE-midazolam) (*n* = 4). **b** Area under the concentration-time curve (AUC) values for the test solutions. *P = 0.047: analyzed using one-way ANOVA with Tukey’s post-hoc multiple comparisons test

The time courses of the changes in the plasma midazolam concentration seen after the oral administration of the test solutions (midazolam solution, LE-midazolam solution, or PEG-LE-midazolam solution) to rabbits are shown in Fig. 2a. Figure 2b shows the AUC for the test solutions. The AUC of the LE-midazolam solution was significantly higher than that of the midazolam solution (*P* = 0.047). However, there was no difference between the AUC values of the PEG-LE-midazolam and midazolam solutions.

### Plasma phosphatidylcholine concentration after the oral administration of the test solutions

**Fig. 3:**
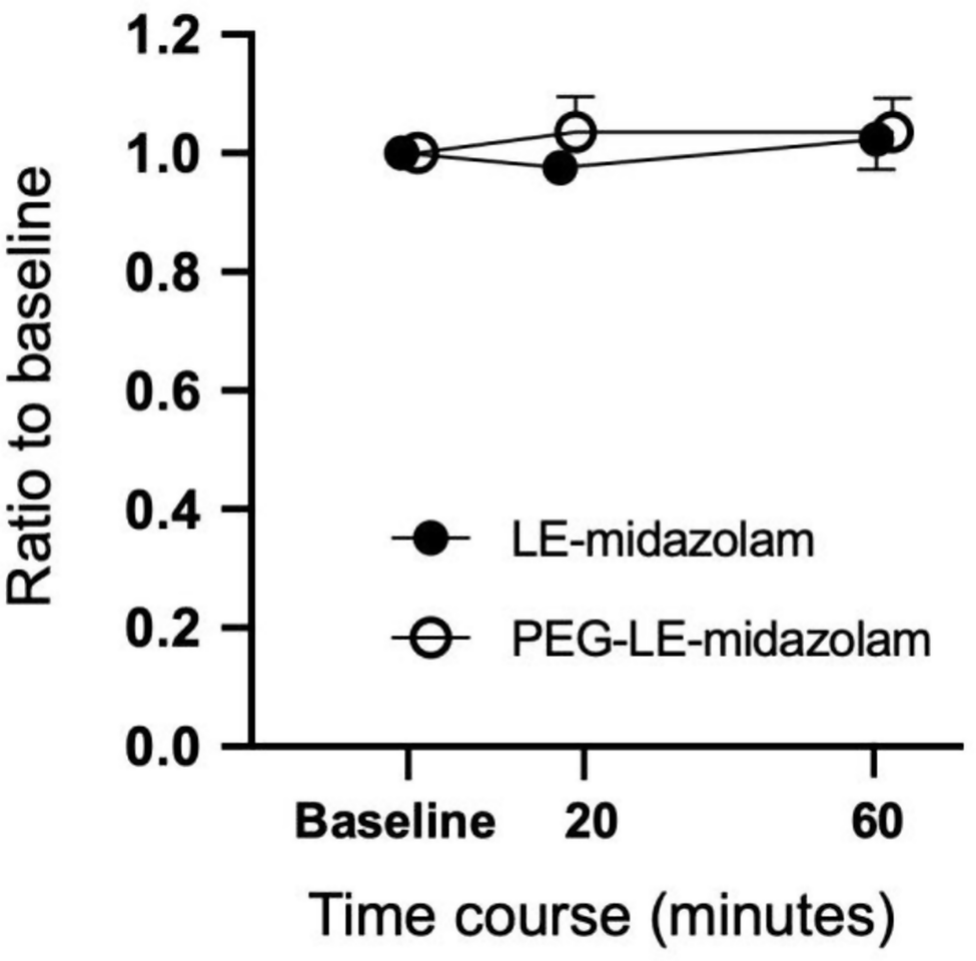
Plasma phosphatidylcholine concentrations seen after the oral administration of the test solutions. The values are expressed as ratios to those seen at the baseline (before the administration of the test solutions). LE-midazolam: liposome-encapsulated midazolam solution (*n* = 4). PEG-LE-midazolam: PEGylated liposome-encapsulated midazolam solution (*n* = 4)

The time courses of the changes in the plasma phosphatidylcholine concentration seen after the oral administration of LE-midazolam or PEG-LE-midazolam solution to rabbits are shown in Fig. 3 as ratios to that seen at the baseline (before the administration of the test solution). The phosphatidylcholine concentrations observed after the administration of the test solutions were not significantly different from those seen at the baseline in either the LE-midazolam or PEG-LE-midazolam solution-treated rabbits.

### Plasma Midazolam concentration after the intravenous administration of the test solutions

**Fig. 4:**
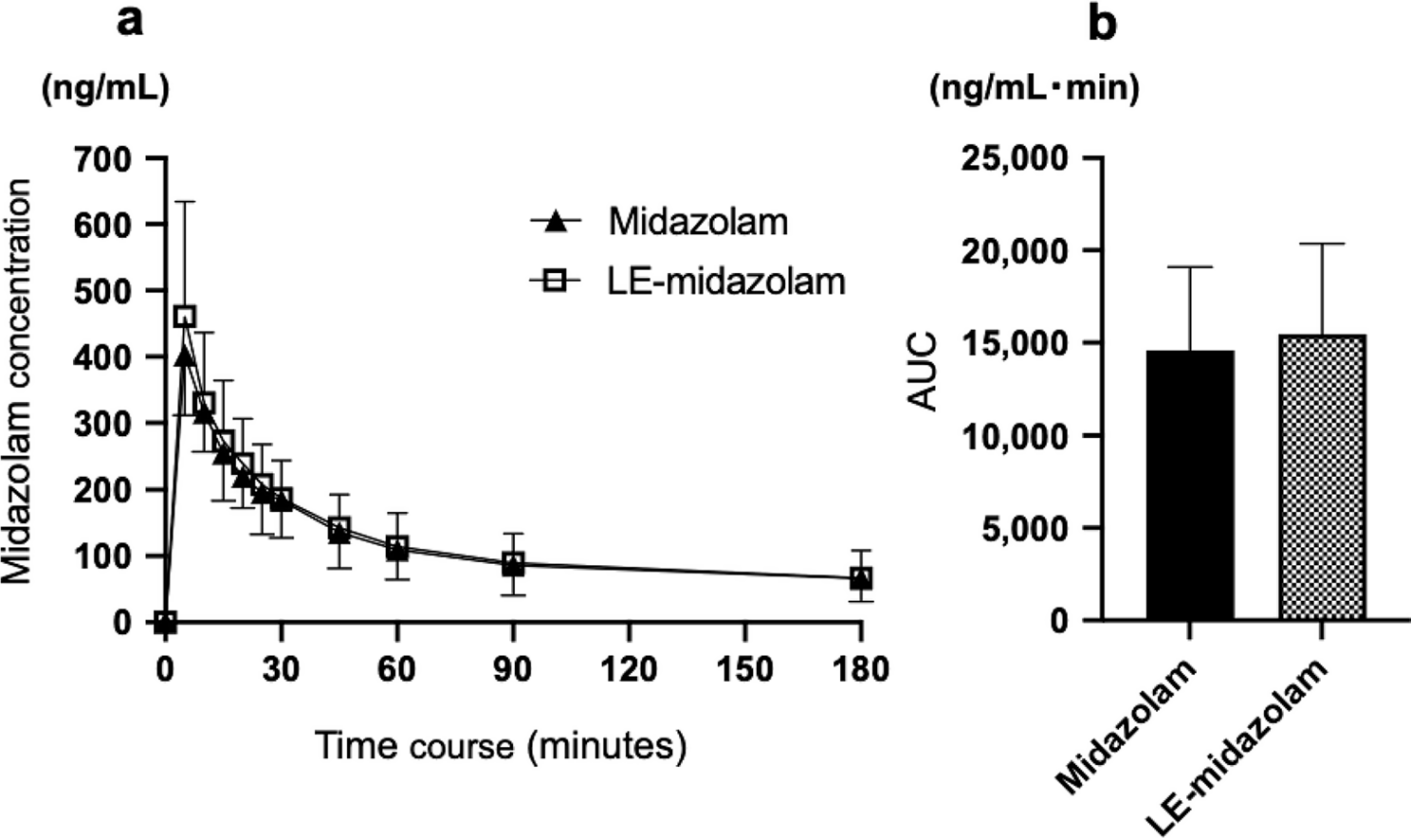
Plasma midazolam concentrations seen after the intravenous administration of the test solutions. **a** Time courses of the changes in the plasma midazolam concentration seen after the intravenous administration of midazolam (*n* = 6) or liposome-encapsulated midazolam solution (LE-midazolam) (*n* = 6). **b** Area under the concentration-time curve (AUC) values of the test solutions

The time courses of the changes in the plasma midazolam concentration seen after the intravenous administration of midazolam solution or LE-midazolam solution to rabbits are shown in Fig. 4a. Figure 4b shows the AUC values for both solutions. There was no difference between the AUC values for the two solutions.

### Bioavailability of the test solution after the oral administration

The average AUC value of plasma midazolam concentration up to 180 min after intravenous administration of midazolam was 14,569.3 ng/mL·min. The bioavailability of the midazolam solution, LE-midazolam solution, and PEG-LE-midazolam solution were 10.0 ± 0.7%, 16.2 ± 4.6%, and 11.7 ± 2.0%, respectively. The bioavailability of the LE-midazolam solution was significantly higher than that of the midazolam solution (*P* = 0.047).

## Discussion

There are few studies about the oral administration of liposomes, and the mechanisms responsible for liposome absorption and distribution in the intestinal tract after the oral administration of liposomes remain unclear [14]. When administered orally, only 50% of the dose of midazolam reaches the circulatory system due to first-pass effects in the liver and intestinal tract [15], and its bioavailability in humans was reported to range from 24 to 46% [16]. The human small intestine is rich in CYPs, including CYP3A, and is reported to be closely involved in the metabolism of midazolam [17]. CYP3A is found in the small intestines of rabbits and metabolizes midazolam in the same manner as in humans [18]. However, when the drug was orally administered in the present study, feed retained in the rabbit intestines may have affected the absorption of the drug, resulting in variations in the experimental results. Among the nutrients present in the feed given to rabbits, fiber requires the longest time to be digested; therefore, we decreased the amount of dietary fiber. AIN93M (Oriental Yeast Co., Ltd.) is the standard feed for rabbits. We specifically ordered AIN93M in which cellulose had been replaced with alfalfa (dietary fiber: 30.0% or less) and gave it to the rabbits from 3 days before the experiment. As a result, the measured blood midazolam concentrations were relatively stable in the present study. However, our results showed lower bioavailability in all test solutions compared to the human bioavailability reported previously [16]. This may be due to differences in the digestive tract environment between humans and rabbits.

In the current study, the oral administration of the LE-midazolam solution led to a higher plasma concentration and bioavailability of midazolam than the oral administration of midazolam solution, which is consistent with the findings of our previous study [5]. It was reported that the activity of CYP in the liver is affected by the lipids found in liposomes, especially phospholipids [19–22]. Therefore, it is possible that liposome encapsulation may partially reduce the first-pass effect in the liver and/or intestinal tract following oral drug administration. Furthermore, because liposomes are taken up into intestinal epithelial cells by endocytosis [23], LE-midazolam may be absorbed better in the intestinal tract. Liposomes are caught by the RES of the liver and spleen [9, 24]. However, since the intravenous administration of LE-midazolam did not increase the plasma midazolam concentration compared with that seen after the intravenous administration of midazolam solution in the present study, which is consistent with a previous study [5], the orally administered liposomes may have affected CYP activity in the intestinal tract, but not in the liver.

The stability of liposome formulations under the strong acidic conditions of gastric acid when administered orally needed to be confirmed. Therefore, we conducted an additional study focusing on the stability of liposomes in such conditions. Specifically, we investigated the changes in midazolam-encapsulating efficiency of liposomes without PEG (LE-midazolam solution) and PEGylated liposomes (PEG-LE-midazolam solution) in a 0.1 N hydrochloric acid solution (approximately pH 1.0) for 30 min (10 min of incubation followed by 20 min of ultracentrifugation). The results showed that both types of liposomes experienced a decrease in midazolam encapsulation, with non-PEG liposomes and PEGylated liposomes decreasing to 31.8 ± 0.9% and 39.6 ± 1.8%, respectively. While PEGylated liposomes were more stable than non-PEG liposomes under these conditions, the bioavailability of the PEG-LE-midazolam solution was still lower than that of the LE-midazolam solution. Therefore, these findings support our conclusion that PEGylation does not enhance the bioavailability of midazolam when administered orally, despite improving the stability of liposomes under acidic conditions.

In our previous study [5], the release and the stability study of liposomal formulations had been already conducted and reported. That study showed that about 80% of the midazolam was released after 30 min from midazolam-encapsulating liposomes sustained in 0.1 N hydrochloric acid solution, representing the stomach, in vitro, and midazolam remained stably encapsulated by liposomes for 1 week in 0.2 M of Tris- hydrochloride buffer solution. However, regarding whether liposomes are absorbed from the intestinal tract in their intact form when administered orally, a perfusion experiment in the rabbit ileum by Patel et al. [25] demonstrated that some liposomes are degraded in the intestinal lumen, but are absorbed intact into intestinal mucosal cells and degraded within these cells. Furthermore, the latter study [25] suggested that liposomes are not released into the bloodstream in an intact form after being taken up into intestinal mucosal cells. The present study showed that the plasma phosphatidylcholine concentrations seen after the administration of the test solutions did not differ significantly from those seen before the administration of the test solutions in either the LE-midazolam or PEG-LE-midazolam solution-treated rabbits. This suggests that the liposomes in both of these solutions were degraded within the intestinal mucosal cells and not released into the circulation, in agreement with the findings of Patel et al. [25].

The effect of microparticle size on absorption into intestinal mucosal cells was similarly demonstrated in another study [26] using the human colon adenocarcinoma cell line Caco-2, which showed that particles with the smallest diameter (100 nm) were absorbed into Caco-2 cells in significantly greater numbers than particles with larger diameters. In in vivo studies, although Shenfield GM et al. [6]. reported that larger liposomes had a greater effect on the bioavailability of the encapsulated drug (insulin) than smaller liposomes, Ong SG et al. [8]. conversely reported that small liposomes increased the bioavailability of the encapsulated drug (griseofulvin). Our study did not demonstrate whether the liposomes were absorbed intact from the intestinal tract, but in an additional study larger liposomes that had not been subjected to homogenization or fine granulation produced lower blood levels of midazolam than smaller liposomes that had been subjected to fine granulation, suggesting that smaller liposomes are more readily absorbed intact by intestinal mucosal cells.

On the other hand, contrary to our hypothesis that PEGylation promotes escape from the first-pass effect in the small intestine and reduces capture by the RES to some extent, there was no difference in the plasma midazolam concentration between orally administered PEG-LE-midazolam solution and orally administered midazolam solution. This suggests that PEGylation did not reduce the metabolism of encapsulated midazolam in the liver or in the intestinal tract. Why did PEGylation not work? In the present study, PEGylation caused no reduction in the midazolam-encapsulating efficiency of the liposomes, although some studies have found that PEGylation reduces the drug-encapsulating efficiency of liposomes [27, 28]. However, the PEG-LE-midazolam solution contained many liposomes of greater than 1,000 nm in diameter, which accounted for about 50% of the liposomes, while in the LE-midazolam solution relatively large liposomes (around 400 nm in diameter) only accounted for about 10% of the liposomes. Particles larger than 100 nm have been reported to show poor uptake in the intestinal tract [29]; therefore, many of the PEGylated liposomes may not have been absorbed from the intestinal tract, resulting in lower bioavailability of the drug after its oral administration than was seen for non-PEGylated liposomes. The reason for the larger size of the PEGylated liposomes could be explained by the difference between the zeta potentials of the liposomes in the PEG-LE-midazolam and LE-midazolam solutions.

The hydrophilic group of phosphatidylcholine consists of an ester bond between a negatively charged phosphate group and a positively charged choline group, keeping it electrically neutral [30]. Although the detailed mechanism is not clear, it is believed that the direction of the ester bond may change in relation to the phase transition temperatures of various phospholipids, resulting in a charged liposomal surface [30]. The stability of the dispersion of liposome particles in liposome solution is known to be affected by the charge state (zeta potential) of the liposomes. As the absolute value of the zeta potential of the particles increases, the repulsive force between the particles becomes stronger, and the dispersion of the molecules becomes more stable [31], resulting in them being less likely to aggregate. In the present study, the zeta potential of the liposomes in the PEG-LE-midazolam solution was closer to zero than that of the liposomes in the LE-midazolam solution. Thus, it is considered that the liposomes in the PEG-LE-midazolam solution were more likely to aggregate. As shown in Fig. 1, LE-midazolam and PEG-LE-midazolam exhibited almost the same particle size at peak 1, indicating that the particle size was almost the same due to homogenization under the same conditions. However, about 50% of the liposomes in the PEG-LE-midazolam solution had a larger particle size (peak 2), indicating that the liposomes in the PEG-LE-midazolam solution had aggregated. In fact, electron microscopic images of the produced liposomes showed numerous PEGylated liposomes that had aggregated (Supplementary Fig. 1). It is possible that orally administered midazolam-encapsulating liposomes aggregated due to PEGylation, making it difficult for the PEGylated liposomes to be absorbed through the gastrointestinal mucosa. Therefore, it is considered that PEGylation may be less effective when a drug is administered orally.

PEG has been reported to cause aggregation [32, 33], and Ogihara et al. [33] demonstrated that liposomes were more likely to aggregate as the molecular weight or concentration of PEG increased. But, in contrast, it was reported to prevent liposome aggregation [34]. Furthermore, Hong et al. [35] showed that PEGylated liposomes exhibited improved gastrointestinal stability and suggested that it is possible that PEGylated liposomes have more polar liposomal surfaces, preventing liposome aggregation. However, they were just guessing that PEG increased the polarity of the liposomal surface because they did not evaluate the zeta potential of the liposomes. On the other hand, they demonstrated that PEGylation did not affect the size distribution of the liposomes. The difference in liposome size between our study and Hong’s study may have been due to the PEGylation method and/or lipid composition. However, this finding of Hong et al. [35]. suggests that the gastrointestinal absorption of liposomes may be improved if PEGylation does not cause liposome aggregation. Therefore, the PEGylation method and/or lipid composition will need to be investigated further.

## Conclusions

In the present study, we evaluated the effects of PEGylating LE-midazolam on the plasma concentration and bioavailability of midazolam when it was administered orally. The results showed that the AUC of the LE-midazolam solution was significantly higher than that of the midazolam solution. However, there was no difference between the AUC values of the PEG-LE-midazolam and midazolam solutions. These findings suggest that liposome encapsulation partially reduces the first-pass effect in the intestinal tract following oral midazolam administration, but PEGylation is not expected to improve the bioavailability of midazolam when it is administered orally.

## Supplementary Information

Below is the link to the electronic supplementary material.

Supplementary Material 1

## Data Availability

The datasets used and/or analyzed during the current study are available from the corresponding author on reasonable request.

## Abbreviations

ANOVA: One-way analysis of variance
AUC: Area under the concentration-time curve
CYP: Cytochrome P450
DLS: Dynamic light scattering
DMPC: Dimyristoylphosphatidylcholine
HPLC: High-performance liquid chromatography
IS: Internal standard
LE-midazolam: Liposome-encapsulated midazolam
PCHL: L-α-phosphatidylcholine
PEG: Polyethylene glycol
PEG-LE-midazolam: PEGylated liposome-encapsulated midazolam
RES: Reticuloendothelial system
SD: Standard deviation
