# Supplementary material for: PEGylation of liposome-encapsulated midazolam does not improve the bioavailability of midazolam when administered orally
Source: BMC Pharmacol Toxicol. 2025 Oct 15;26:166. doi: 10.1186/s40360-025-00993-1 (PMC12522756; doi:10.1186/s40360-025-00993-1)
Supplement: Supplementary file 1 — Supplementary Material 1 [file 40360_2025_993_MOESM1_ESM.docx]

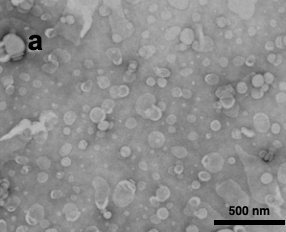


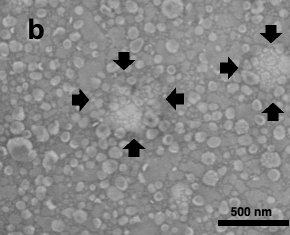


**Supplementary Fig. 1**

Electron microscopy images (a) of liposome particles without PEG and (b) of PEGylated liposome particles. The aggregated liposomes are surrounded by arrows.
